# Consequences of the Poor Anticoagulation Control of Patients with Non-Valvular Atrial Fibrillation Treated with Vitamin K Antagonists

**DOI:** 10.3390/jcm13216495

**Published:** 2024-10-30

**Authors:** Antoni Sicras Mainar, Joel Salazar-Mendiguchía, María Isabel del Campo Alonso, Ainara Echeto, David Vilanova Larena, Josep Comín Colet

**Affiliations:** 1 Atrys Health, 28021 Madrid, Spain; 2 Bristol Myers Squibb, 28050 Madrid, Spain; joel.salazar@bms.com (J.S.-M.); mariaisabel.campoalonso@bms.com (M.I.d.C.A.); ainara.echeto@bms.com (A.E.); david.vilanovalarena@bms.com (D.V.L.); 3 Cardiology Department, Bellvitge University Hospital, L’Hospitalet de Llobregat, 08907 Barcelona, Spain; josepcomin@gmail.com; 4 IDIBELL—Instituto de Investigación Biomédica de Bellvitge, L’Hospitalet de Llobregat, 08908 Barcelona, Spain; 5 CIBERCV—Centro de Investigación Biomédica en Red Enfermedades Cardiovaculares, Universitat de Barcelona, Hospitalet del Llobregat, 08007 Barcelona, Spain

**Keywords:** anticoagulants, atrial fibrillation, health resources, healthcare costs, hospitalization

## Abstract

**Background:** The prevention of thromboembolisms through anticoagulation and heart rate control is crucial in managing non-valvular atrial fibrillation (NVAF). This study aimed to analyze the consequences of poor anticoagulation control with vitamin K antagonists (VKAs) in Spanish patients with NVAF, focusing on thrombotic events, bleeding, mortality, healthcare resources (HRU), and costs. **Methods:** This observational, retrospective study used electronic medical records (BIG-PAC^®^ database) of NVAF patients who started VKA treatment between 1 January 2016 and 31 December 2018. Patients were followed up for two years and classified by poor or adequate anticoagulation control. Demographic and clinical characteristics, treatments, incidence of cardiovascular events, mortality rates, HRU, and costs were analyzed. **Results:** Patients with poor control (*n* = 2136) had a 75% greater probability of suffering a cardiovascular event compared to patients with adequate control (*n* = 2351) (HR, 1.75 [95%CI: 1.43–2.14; *p* < 0.001]). Cardiovascular events, major bleeding, minor bleeding, systemic thromboembolism, and ischemic strokes were reduced by 32.1%, 46.2%, 29.6%, 22.2%, and 16.1%, respectively. It was estimated that adequate anticoagulant control saved EUR 455/patient with NAVF due to reduced hospitalization for cardiovascular events. **Conclusions:** For VKA-treated NVAF patients, poor anticoagulation control was associated with a higher number of cardiovascular events, greater consumption of HRU, and higher management costs than for patients with adequate control.

## 1. Introduction

Atrial fibrillation (AF) is the most common cardiac arrhythmia, characterized by uncoordinated atrial activation, leading to ineffective contraction [1,2]. AF is a disabling condition responsible for up to 30% of strokes [3]. Additionally, AF patients face a significantly increased risk of mortality due to sudden cardiac death, heart failure, or stroke [3]. According to the OFRECE study, the prevalence of AF in Spain is 4.4% in people aged > 40 years [4], and around 80% of them suffer non-valvular AF (NVAF) [5,6]. The primary focus of NVAF management is the prevention of thromboembolisms and effective heart rate control [3].

Vitamin K antagonists (VKAs), like acenocoumarol and warfarin, have been widely used to prevent cardioembolic complications in NVAF patients [2,7,8,9]. Patients receiving VKA therapy require regular monitoring of their prothrombin time, usually expressed as the International Normalized Ratio (INR). It evaluates the risk of bleeding or patient coagulation status and allows for adjustment of the doses of VKA [10]. The INR value is dimensionless and varies between 2.0 and 3.0 [10,11]. The use of VKAs is subject to high interpatient variability, and external factors such as diet, weight changes, diseases, and concomitant medications may influence the coagulation statuses of patients [12,13,14,15]. Patients have poor anticoagulation control if, at least during the first 6 months, their time in the therapeutic range (TTR) is determined to be lower than 65% using the method developed by Rosendaal or lower than 60% according to the direct method [16]. It is estimated that 39.4% to 57.2% of patients receiving VKAs have poor anticoagulation control according to the Rosendaal method [17,18,19,20].

Poor anticoagulation control has serious clinical implications, including an increased risk of cardiovascular events such as stroke, systemic embolism, major bleeding, acute coronary syndrome, and heart failure and even death compared to patients with adequate control [21,22,23,24]. It was reported that there is no difference in anticoagulation control between acenocoumarol and warfarin [25]. Managing complications arising from poor anticoagulation control also imposes a substantial economic burden [18,26,27]. In Spain, an analytical model developed by Barrios et al. estimated that the incremental costs of managing this patient population amounted to about EUR 30 million from the Spanish National Health System (SNHS) perspective and EUR 75 million from a societal perspective [18].

There is a lack of information about the clinical consequences of poor anticoagulation control in patients with NVAF, the subsequent consumption of healthcare resources, and the management costs for the SNHS obtained using real-life data. Thus, this study aims to analyze the impact of poor anticoagulation control using VKAs on thrombotic events, minor and major bleeding, and mortality among NVAF patients. Additionally, the uses of healthcare resources for NVAF patients with adequate or poor control, including visits to primary care physicians and nurses, visits to specialized care, hospitalizations, and associated healthcare costs, were also estimated.

## 2. Materials and Methods

### 2.1. Study Design and Data Collection

This is an observational, retrospective study based on the electronic medical records (EMRs) of the BIG-PAC^®^ database, which includes anonymized medical records of 1.8 million Spanish patients from the time of the study, representing 4–5% of this population. It contains data from general practitioner visits, emergency care, prescriptions, hospital admissions, etc., from primary care centers and hospitals in seven autonomous communities in Spain [28]. These EMRs underwent rigorous anonymization in the centers/hospitals of origin in compliance with Organic Law 3/2018 of 5 December on the Protection of Personal Data and the guarantee of digital rights [29]. The BIG-PAC^®^ database proved to be representative of the Spanish population [30].

### 2.2. Study Population

The study population consisted of patients with NVAF who started their treatment with VKAs (acenocoumarol or warfarin) between 1 January 2016 and 31 December 2018. The index date was the date of initiating the VKA treatment, and patients were followed up to 2 years from the index date. The end of follow-up was defined as completion of two years of follow-up, patient disenrollment from the database, treatment discontinuation, or death (whichever occurred first).

The diagnoses were obtained using the International Classification of Diseases, 10th Edition, Clinical Modification (ICD-10-CM) (Table S1) [31].

Inclusion criteria were (a) being ≥18 years old, (b) having a diagnosis of AF in addition to starting oral anticoagulation treatment with VKAs between 2016 and 2018, (c) being active in the database for ≥12 months before this study’s initiation, (d) being included in the chronic prescription program (with documentation of daily dose, time intervals, and treatment duration for each administered medication, as well as at least two prescriptions during the follow-up period), and (e) being regularly monitored (with at least two records in the computer system).

Exclusion criteria were (a) having been diagnosed with AF with mitral valve heart disease; (b) having been transferred to other sites, displaced or out of the area; (c) being permanently institutionalized; (d) having a history of AF secondary to reversible causes (thyrotoxicosis, pericarditis), undergoing heart surgery, experiencing venous thromboembolism, or having undergone hip or knee surgery in the previous 6 weeks, having valvular heart disease, or being pregnant; (e) having valvular AF (with mechanical heart valve or moderate-severe mitral stenosis); and (f) having end-stage kidney disease, undergoing dialysis, or having had a kidney transplant.

#### Study Cohorts

Two cohorts were defined: (1) patients with poor anticoagulation control and (2) those with adequate control. The first group of patients were those with a TTR level < 65% (Rosendaal method) or a TTR level < 60% (direct method) in the first 6 months of the treatment with VKAs [16]. The second group included those who did not meet this criterion.

### 2.3. Study Variables

#### 2.3.1. Sociodemographic Characteristics and Comorbidities

The sociodemographic characteristics and the prevalence of comorbidities of the study population were estimated at the index date. The sociodemographic characteristics included age and sex, smoking status, and the consumption of alcohol. The body mass index (BMI) was also considered. Comorbidities included hypertension, diabetes, dyslipidemia, obesity, myocardial infarction, hemorrhagic and ischemic stroke, transient ischemic attack, peripheral artery disease, cardiac insufficiency, renal insufficiency, asthma, chronic obstructive pulmonary disease, dementia, depression, neoplasm, and hepatic insufficiency. The Charlson comorbidity index was used as a summary variable of general comorbidity and an approximation to the severity levels of the patients [32].

#### 2.3.2. Pharmacological Treatment

The data used came from drug-dispensing records, and the corresponding prescriptions were made at the physicians’ discretion, according to clinical practice. Drugs were coded using the Anatomical Therapeutic Chemical Classification System (ATC) [33], including VKAs (acenocoumarol [code: B01AA07] and warfarin [code: B01AA03]). Data on the time from NVAF diagnosis, as well as the prescribed treatment, were collected.

Treatment persistence/duration of the anticoagulant therapy was estimated from the index date (start date) to 2 years, the development of a new event (hemorrhagic/ischemic stroke, bleeding), the switch to another antiplatelet/anticoagulant treatment other than that which motivated the patient’s inclusion in the succeeding 30 days, the abandonment of treatment (≥60 days without renewing the medication), or death, whichever occurred first. Treatment persistence was obtained at 12- and 24-month follow-ups. The date of interruption was 30 days from the date of the last prescription.

The concomitant treatment, defined as the drugs administered in combination with the anticoagulation treatment, was described at the index date and the end of the follow-up period. These drugs were classified as non-steroidal anti-inflammatory drugs (NSAIDs) (code: M01AE), antiplatelets (code: B01AC), antidiabetics (code: A10), beta-blockers (code: C07), agents acting on the renin–angiotensin system (code: C09), and lipid-lowering agents (code: C10).

#### 2.3.3. Cardiovascular Events and Mortality

The consequences of the poor anticoagulation control were estimated based on the cardiovascular events that occurred during the follow-up period, including thromboembolic events (including ischemic stroke and systemic thromboembolism) and major and minor bleedings.

Major bleeding included bleeding in intracranial, gastrointestinal, and other locations (liver, eye, and spleen). These events were defined as bleeding requiring hospital admission, which is defined as acute or subacute bleeding accompanied by ≥1 of the following criteria: (a) reduction in hemoglobin levels of ≥2 g/dL, (b) transfusion of ≥2 red blood cell concentrates, and (c) fatal bleeding (intracranial, intraspinal, intraocular, pericardial, intraarticular, intramuscular with compartmental syndrome, retroperitoneal). Minor bleeding consisted of bleeding events that did not meet these criteria. Major and minor types of bleeding were identified using the ICD-10-CM codes (Table S1) from 30 days after initiation of anticoagulant therapy (acenocoumarol or warfarin) until the date of treatment discontinuation. These events were also identified 6 months before the index date (baseline period).

#### 2.3.4. CHA_2_DS_2_-VASc and HAS-BLED Scores

The congestive heart failure, hypertension, age ≥ 75 (doubled), diabetes, stroke (doubled), vascular disease, age 65 to 74, and sex category (female) (CHA_2_DS_2_-VASc) score was used to estimate the risk of stroke in the study population (Table S2). In contrast, hypertension, abnormal renal/liver function, stroke, bleeding history or predisposition, labile INR, elderly, and use of drugs/alcohol concomitantly (HAS-BLED) score was used to evaluate the bleeding risk (Table S3) [34]. The CHA_2_DS_2_-VASc and HAS-BLED scores were measured during the follow-up period [34].

#### 2.3.5. Mortality

The mortality rate at the end of the study (the percentage of patients who died during the follow-up period) was also estimated.

#### 2.3.6. Healthcare Resources and Healthcare Costs

The use of healthcare resources and the healthcare costs associated with treating and monitoring the patients included in this study during the 2-year follow-up period were estimated. Healthcare resources included medical visits (primary care, nursing, specialist care [cardiology, internal medicine, endocrinology, vascular, neurology, hematology, geriatrics], hospital emergencies), hospitalizations, and drugs. In addition, diagnostic/therapeutic tests included laboratory tests, conventional radiology, computed tomography, magnetic resonance, catheterization, angioplasty, and endarterectomy/thrombectomy. The number and percentage of patients in each cohort who used these healthcare resources were estimated.

Costs were expressed in 2021 Euros [35] as the mean cost per patient (mean/unit). They were estimated as the use of healthcare resources multiplied by the unit cost of each resource (Table S4). Drug costs were calculated by using the retail price per pack at the time of prescription (according to Bot Plus, General Council of Colleges of Official Pharmacists of Spain [36]) and the use of drugs according to the dispensing records.

### 2.4. Statistical Analysis

The database search criteria were based on computer statements (SQL scripts). The data were carefully reviewed and prepared for analysis by observing the frequency distributions and searching for possible recording or coding errors.

Descriptive univariate statistical analyses were performed; qualitative data were described using absolute and relative frequencies (*N*, %), and quantitative variables were described using means and standard deviations (SDs) for symmetric distributions, and medians and interquartile ranges (IQR, P25–P75; Q1–Q3) for asymmetric distributions. In addition, 95% confidence intervals (CIs) were calculated for population parameters.

Bivariate analyses were developed to compare incident cases; ANOVA and chi-squared tests were used for independent groups. Patient age, sex, and Charlson comorbidity index values were used as covariables to adjust cohort differences. These statistical analyses allowed us to compare the demographic variables, comorbidities, and medication administered between cohorts. Cox proportional hazard risk models were used to estimate the time until the first cardiovascular event occurred., including thromboembolic and bleeding events. Percentage results were obtained, equivalent to one ratio per 100 people-year (incidence rate; accumulated risk). The data were censored in the absence of an event. An analysis of covariance (ANCOVA; generalized linear model; estimate of marginal means; Bonferroni adjustment) was used to correct costs when independent groups were compared. The statistical program SPSSWIN version 27 was used, and values of *p* < 0.05 were considered statistically significant.

## 3. Results

### 3.1. Study Population

**Figure 1:**
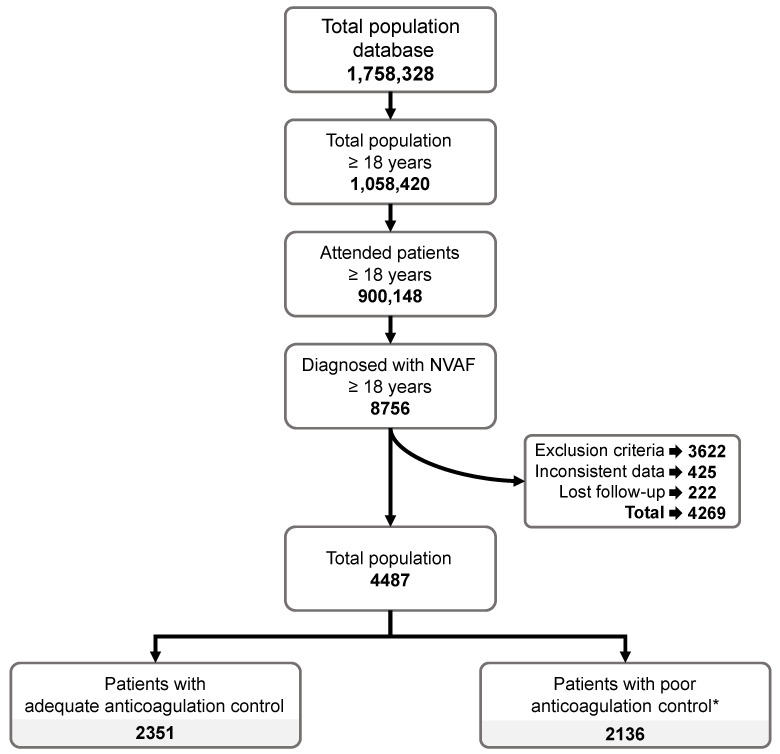
Patient flow chart. * Poor anticoagulation control was defined as a TTR level of <65% (determined via the Rosendaal method) or a TTR level of <60% (determined via the direct method).

This study considered an attended population of 900,147 patients aged 18 years and above, of which 8756 were diagnosed with NVAF (Figure 1). Regarding the inclusion and exclusion criteria, after discarding records with inconsistent data and those of patients lost to follow-up, 4487 patients were finally considered in this study. The patients were categorized into two groups: 2351 with adequate anticoagulation control and 2136 with poor anticoagulation control (Figure 1).

#### Characteristics of the Study Population

The patients with poor anticoagulation control were older than those with adequate control (70.6 years [SD: 7.9] vs. 69.5 years [SD: 11.4]; *p* < 0.001). However, no differences in terms of sex were found between both groups (Table 1).

Overall, the NVAF patients with poor anticoagulation control had more comorbidities and a higher Charlson comorbidity index than the NVAF patients with adequate anticoagulation (*p* < 0.001 in both comparisons). However, the frequencies of each comorbidity were similar in both groups, except for peripheral artery disease, which was more frequent in those with poor anticoagulation control (*p* < 0.002) (Table 1).

It should be noted that the prevalence of cardiovascular comorbidities such as hemorrhagic/ischemic strokes was similar in both cohorts, and both groups had had a similar incidence of major bleeding during the baseline. However, the NVAF patients with poor control had had more minor bleeding events than those with adequate anticoagulation control (*p* < 0.003). At baseline, the patients with poor anticoagulation control had a higher risk of suffering cardiovascular events and bleeding compared to those with adequate anticoagulation control (CHA2DS2-VASc scores: 2.6 [SD: 1.6] vs. 3.4 [SD:1.2]; HAS-BLED scores: 2.8 [SD: 1.0] vs. 3.4 [SD: 0.7]; *p* < 0.001 for both comparisons) (Table 1).

### 3.2. Cardiovascular Events and Mortality

**Figure 2:**
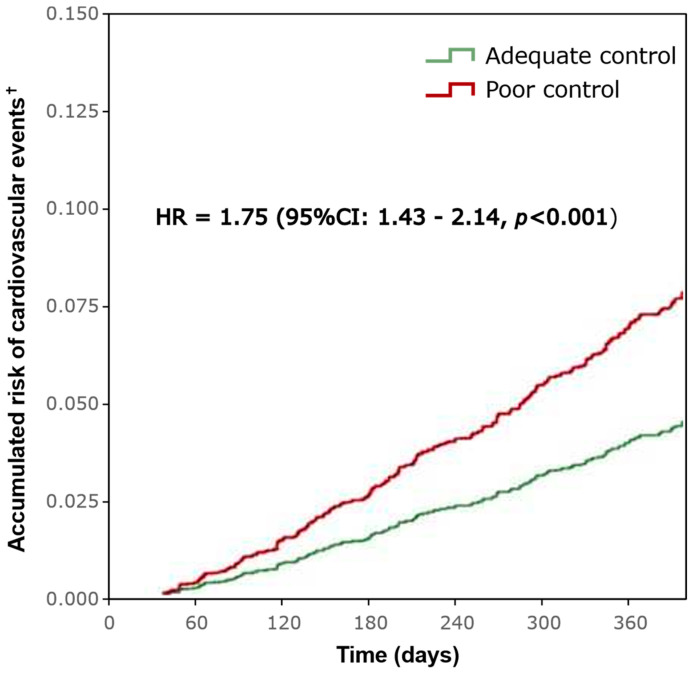
Event-free progression analysis. ^†^ Cardiovascular events include major and minor bleeding, ischemic stroke, and systemic thromboembolism.

Cardiovascular events were more frequent during the follow-up for patients with poor anticoagulation control than for those with adequate control (30.5% vs. 20.7%; reduction: 32.1%; *p* < 0.001). The average number of events during the follow-up period was also higher for patients with poor control (0.3 events [SD: 0.5] vs. 0.2 events [SD: 0.5]; *p* < 0.001) (Table 2). Therefore, it was estimated that patients with poor anticoagulation control had a 75% greater risk of suffering cardiovascular events in comparison to patients with adequate control (*HR*: 1.75, 95% CI: 1.43–2.14, *p* < 0.001) (Figure 2).

Most cardiovascular events were minor bleeding (17.2%), followed by major bleeding (7.0%) and ischemic strokes (2.8%). The highest reductions in patients with poor control vs. adequate control were observed in major bleeding events (46.2%), minor bleeding events (29.6%), systemic thromboembolism (22.2%), and ischemic strokes (16.1%). The mortality rate was also higher for patients with poor control than for those with adequate control (10.8% vs. 7.3%; *p* < 0.001) (Table 2).

#### Variations in CHA_2_DS_2_-VASc and HAS-BLED Scores

Table 3 shows the variations in the CHA_2_DS_2_-VASc and HAS-BLED scores at the index date and the end of the study. In general, the patients with adequate anticoagulation control had a significantly lower risk of cardiovascular events and bleeding than those with poor control, both at the start of the study and at the end of the follow-up. Throughout this study, both groups experienced elevations in their cardiovascular and bleeding risk scores. However, the poor control group demonstrated a significantly greater increase in these measures than the adequate control group (Table 3).

### 3.3. Treatment of the Study Population

#### 3.3.1. Anticoagulant Treatment

The times from diagnosis to the first prescription were similar in both study cohorts (12.8 years [SD: 23.3]). Still, the duration of the treatment was longer for those with adequate control compared to that for those with poor anticoagulation control (576 days vs. 355 days, *p* < 0.001) (Table 4).

Acenocoumarol was the most frequently prescribed anticoagulant drug (90.2%), and there were no differences between the study cohorts. For patients with adequate control, the longest treatment was observed for those receiving acenocoumarol (579.5 days vs. 564 days). In contrast, for those with poor control, the longest treatment was reported for patients treated with warfarin (376.5 days vs. 354 days) (Table 4).

The persistence of the anticoagulant therapy was higher for patients with adequate control than for those with poor control (at 12 months: 57.8% vs. 49.3%; at 24 months: 43.8% vs. 34.9%; *p* < 0.001 in both comparisons). The main causes of discontinuation were the incidence of new events (25.5%) and a switch of medication (15.4%) (Table 4).

#### 3.3.2. Variations in Concomitant Medication

Concomitant medication mainly consisted of agents acting on the renin–angiotensin system, followed by NSAIDs, beta-blockers, and lipid-lowering agents. The values regarding the use of concomitant drugs were similar in both study cohorts at the index date and the end of the study. However, patients with poor anticoagulation control received more lipid-lowering agents than those with adequate control. Overall, there was an increase in the consumption of concomitant medication between the index date and the end of the study (*p* < 0.001 in all comparisons). These increases were particularly relevant in terms of the consumption of NSAIDs (*p* < 0.013), antidiabetics (*p* < 0.001), beta-blockers (*p* = 0.041), and lipid-lowering agents (*p* < 0.001) (Table 3).

### 3.4. Use of Healthcare Resources and Costs

Patients with poor anticoagulation control required more visits to primary care physicians *(p* = 0.019), nurses (*p* < 0.001), and specialists (*p* = 0.002) in comparison to patients with adequate control. In addition, the former required more and longer hospitalizations during the follow-up period than the patients with adequate control (*p* < 0.001 in both comparisons) (Table 5).

Overall, there was no difference in test prescription between the study cohorts, except for laboratory testing. The poor control group required significantly more laboratory tests than the adequate control group (0.06 [SD:0.46] vs. 0.15 [SD:0.76]; *p* < 0.001) (Table 5).

The management costs for these patients amounted to EUR 2232 (SD: 2340), being higher for the patients with poor control (EUR 2477 [SD: 2554]). After the adjustment for the characteristics of patients, it was observed that having adequate anticoagulation control saved EUR 455 per patient. The most important cost categories were hospitalizations (EUR 901/patient), followed by primary care visits (EUR 375/patient) and nurse visits (EUR 265/patient). It should also be noted that patients with poor anticoagulation control had greater costs associated with the concomitant medication compared to those with adequate control (EUR 356/patient vs. EUR 344/patient; *p* < 0.045) (Table 6).

## 4. Discussion

Our results show that patients with NVAF and adequate anticoagulation control had a 32.1% lower rate of suffering cardiovascular events than those with poor anticoagulation control. The main benefits reported were the reductions in minor bleeding, major bleeding, and ischemic strokes. Patients with adequate anticoagulation control also had lower risks of cardiovascular events and bleeding according to the CHA_2_DS_2_-VASc and HAS-BLED scores. On average, the duration of the anticoagulant therapy was around 44 days longer for those with adequate control. Patients with poor anticoagulation control required more healthcare resources, such as visits to primary care physicians, nurses, and specialists, and more and longer hospitalizations in comparison to patients with adequate control. Therefore, it was estimated that having adequate anticoagulation control was associated with a reduction of EUR 455 per patient (18.4%) from the perspective of the SNHS.

In the Spanish context, the characteristics of NVAF patients with poor anticoagulation control despite the treatment with VKA were previously analyzed by Dalmau et al. [25]. They considered a cohort of 41,430 patients, and 41.8% of them had poor anticoagulation control. This was associated with being female and younger than 60 years old. Other associated factors were having previous cardiovascular disorders (peripheral arterial disease, heart failure, ischemic heart disease, a history of intracranial hemorrhage, and gastrointestinal bleeding) and other comorbidities such as diabetes mellitus, renal insufficiency, and liver failure. In addition, they associated poor anticoagulation control with a history of alcoholism. In contrast, we estimated that patients with poor anticoagulation control were older (mean age: 70.6 years), but in line with Dalmau et al., most were women (52.6%). We also observed that patients with poor control had more comorbidities in general, but the prevalences of most of those comorbidities (particularly those associated with the cardiovascular system) were similar at the index date of this study. These variations may be associated with the fact that our study included patients from primary care centers and hospitals of seven autonomous communities. In contrast, the study conducted by Dalmau et al. evaluated the population registered in Catalonia’s System for the Improvement of Research in Primary Care (SIDIAP) database [25]. Therefore, the differences in managing the NVAF population among the Spanish autonomous communities and the characteristics of patients who attended the primary care centers or hospitals may have contributed to these variations. One of the main contributions of our study relates to the analyses of the clinical consequences for this patient population. We estimated that having poor anticoagulation control is associated with the occurrence of more cardiovascular events, such as minor and major bleeding and ischemic strokes.

The economic burden associated with the clinical consequences of poor anticoagulation control in Spain was estimated by Barrios et al. by using a simulation model [18]. They considered a hypothetical population of 594,855 patients, 48.3% of whom had poor anticoagulation control, resulting in 2321 more ischemic strokes, 2236 more major bleeding events, and 14,463 more deaths (representing reductions of 40.0%, 25.7%, and 43.4%, respectively), compared to those with adequate coagulation control. Therefore, they estimated that adequate anticoagulation control would lead to cost savings of EUR 116/patient-year (a reduction of 34.4%), mainly due to the costs of ischemic stroke. These results are similar to ours since we observed that adequate anticoagulation control reduced the frequency of cardiovascular events (32.1%), particularly major bleeding (46.2%), minor bleeding (29.6%), systemic thromboembolism (22.2%), and ischemic strokes (16.1%). Our study shows that adequate anticoagulation control saved EUR 455/patient during the 2-year follow-up period, corresponding to a reduction of 18.4% in the cost per patient. We also observed that most of the cost reductions were associated with a reduction in hospitalizations for patients with adequate anticoagulation control. The variations between our research and the study carried out by Barrios et al. may relate to the fact that their analysis was based on data from clinical trials, whereas ours was based on real-life patients. We used a population of NVAF patients registered in the BIG-PAC^®^ database, which is representative of the Spanish population [30].

Our study has some limitations. First, the BIG-PAC^®^ database is administrative in nature and has some deficiencies when it is used for observational research. Therefore, there may be missing data on the study population, particularly if certain patients attended public or private centers outside of the area of influence of BIG-PAC^®^. Second, there are limitations regarding the categorization of the disease and possible classification bias of patients. In this vein, the ICD-9-CM coding system does not allow for the differentiation of permanent, persistent, and paroxysmal AF. Third, this study did not analyze variables that could influence the results, like the socioeconomic levels of the patients. Finally, a significant limitation of this study is its inability to establish causal relationships based on the data collected. Due to this study’s observational nature, the findings are limited to demonstrating associations between variables rather than establishing direct cause-and-effect relationships. It should be noted that despite these limitations, our results may be of interest with respect to improving the anticoagulation control of patients with NVAF and healthcare decision-making in public healthcare systems.

## 5. Conclusions

Poor anticoagulation control in NVAF patients undergoing treatment with VKA was associated with a higher incidence of cardiovascular events, such as major and minor bleeding, ischemic strokes, and systemic thromboembolism. These patients also required more healthcare resources and had higher management costs than patients with adequate anticoagulation control. Therefore, using other therapeutic alternatives may improve these patients’ clinical outcomes and reduce the economic burden of NVAF for the SNHS.

## Figures and Tables

**Table 1:** Sociodemographic characteristics, comorbidities, and scores.

Study Cohort	Adequate Anticoagulation Control	Poor Anticoagulation Control	Total	*p*-Value
*N* (%)	2351 (52.4)	2136 (47.6)	4487 (100)	
Demographic characteristics				
Age, mean (SD)	69.5 (11.4)	70.6 (7.9)	70.0 (9.9)	**<0.001**
Age range, N (%)				**<0.001**
18–49 years	76 (3.2)	9 (0.4)	85 (1.9)	
50–64 years	547 (23.3)	71 (3.3)	618 (13.8)	
65–74 years	638 (27.1)	709 (33.2)	1347 (30)	
75–84 years	765 (32.5)	944 (44.2)	1709 (38.1)	
≥85 years	325 (13.8)	403 (18.9)	728 (16.2)	
Sex, males, *N* (%)	1129 (48)	1012 (47.4)	2141 (47.7)	0.666
Smoke active, *N* (%)	114 (4.8)	148 (6.9)	262 (5.8)	**0.003**
BMI, kg/m^2^, mean (SD)	29.9 (5.2)	30.1 (5.1)	30 (5.1)	0.157
Comorbidities, mean (SD)	2.7 (1.8)	2.9 (1.4)	2.8 (1.6)	**<0.001**
Comorbidities, *N* (%)				
Hypertension	1675 (71.2)	1534 (71.8)	3209 (71.5)	0.673
Diabetes	697 (29.6)	650 (30.4)	1347 (30)	0.567
Dyslipidemia	1044 (44.4)	1002 (46.9)	2046 (45.6)	0.093
Obesity	543 (23.1)	512 (24)	1055 (23.5)	0.491
Alcoholism	50 (2.1)	51 (2.4)	101 (2.3)	0.556
Myocardial infraction	142 (6)	123 (5.8)	265 (5.9)	0.690
Hemorrhagic stroke	7 (0.3)	4 (0.2)	11 (0.2)	0.455
Ischemic stroke	170 (7.2)	180 (8.4)	350 (7.8)	0.136
Transient ischemic attack	72 (3.1)	79 (3.7)	151 (3.4)	0.238
Peripheral artery disease	128 (5.4)	165 (7.7)	293 (6.5)	**0.002**
Heart insufficiency	433 (18.4)	426 (19.9)	859 (19.1)	0.194
Renal insufficiency	181 (7.7)	186 (8.7)	367 (8.2)	0.218
Asthma	166 (7.1)	176 (8.2)	342 (7.6)	0.137
COPD	392 (16.7)	380 (17.8)	772 (17.2)	0.322
Dementia	55 (2.3)	42 (2)	97 (2.2)	0.391
Depression	203 (8.6)	196 (9.2)	399 (8.9)	0.525
Neoplasm	179 (7.6)	172 (8.1)	351 (7.8)	0.585
Hepatic insufficiency	113 (4.8)	125 (5.9)	238 (5.3)	0.119
Scores				
Charlson comorbidity index, mean (SD)	1.4 (1.4)	1.6 (1.5)	1.5 (1.5)	**<0.001**
Patients with a score of 0, *N* (%)	690 (29.3)	560 (26.2)	1250 (27.9)	**<0.001**
Patients with a score of 1, *N* (%)	796 (33.9)	667 (31.2)	1463 (32.6)	
Patients with a score of 2, *N* (%)	467 (19.9)	441 (20.6)	908 (20.2)	
Patients with a score ≥ 3, *N* (%)	398 (16.9)	468 (21.9)	866 (19.3)	
CHA_2_DS_2_VASc score, mean (SD)	2.6 (1.6)	3.4 (1.2)	3 (1.5)	**<0.001**
Patients with a score of 0, *N* (%)	276 (11.7)	32 (1.5)	308 (6.9)	**<0.001**
Patients with a score of 1, *N* (%)	406 (17.3)	99 (4.6)	505 (11.3)	
Patients with a score of 2, *N* (%)	444 (18.9)	331 (15.5)	775 (17.3)	
Patients with a score ≥ 3, *N* (%)	1225 (52.1)	1674 (78.4)	2899 (64.5)	
HAS-BLED score, mean (SD)	2.8 (1.0)	3.4 (0.7)	3.1 (0.9)	**<0.001**
Patients with a score of 0, *N* (%)	14 (0.6)	0 (0)	14 (0.3)	**<0.001**
Patients with a score of 1–2, *N* (%)	950 (40.4)	48 (2.3)	998 (22.2)	
Patients with a score of 3–5, *N* (%)	1372 (58.4)	2079 (97.3)	3451 (76.9)	
Patients with a score ≥ 6, *N* (%)	15 (0.6)	9 (0.4)	24 (0.6)	
Patients per type of event *, *N* (%)				
Minor bleeding	159 (6.8)	195 (9.1)	354 (7.9)	**0.003**
Major bleeding	129 (5.5)	101 (4.7)	230 (5.1)	0.250

Data are expressed as mean values with standard deviations and percentages. * Events registered during the baseline period, 6 months before the index date. Bold *p* values indicate statistical significance (*p* > 0.05). BMI: Body mass index; COPD: chronic obstructive pulmonary disease; *p*: significance value; SD: standard deviation.

**Table 2:** Cardiovascular events and mortality during the follow-up period.

Study Cohort	Adequate Anticoagulation Control	Poor Anticoagulation Control	Total	*p*-Value
N (%)	2351 (52.4)	2136 (47.6)	4487 (100)	
Cardiovascular events				
For patients with cardiovascular events, N (%)	486 (20.7)	651 (30.5)	1137 (25.3)	**<0.001**
95% CI	(19.1–22.3)	(28.5–32.5)	(24.0–26.6)	
Mean number of events, N (SD)	0.2 (0.5)	0.3 (0.5)	0.3 (0.5)	**<0.001**
Number of events, N (%)				
Patients with 1 cardiovascular event	440 (18.7)	589 (27.6)	1029 (22.9)	
Patients with ≥ 2 cardiovascular events	46 (2.0)	62 (2.9)	108 (2.4)	**<0.001**
Type of event, N (%)				
Ischemic strokes	62 (2.6)	67 (3.1)	129 (2.8)	**0.025**
Systemic thromboembolisms	16 (0.7)	21 (0.9)	37 (0.8)	**0.003**
Minor bleeding	337 (14.3)	434 (20.3)	771 (17.2)	**<0.001**
Major bleeding	118 (5.0)	198 (9.3)	316 (7.0)	**<0.001**
Mortality, N (%)	172 (7.3)	231 (10.8)	404 (9)	**<0.001**
95% CI	(6.2–8.4)	(9.5–12.1)	(8.2–9.8)	

Data are expressed as mean values with standard deviations and percentages. Bold *p* values indicate statistical significance (*p* > 0.05). CI: confidence interval; *p*: statistical significance; SD: standard deviation.

**Table 3:** Variations between the index date and the end of the study in the scores and concomitant medication.

Study Cohort	Adequate Anticoagulation Control	Poor Anticoagulation Control	Total	*p*-Value
N (%)	2351 (52.4)	2136 (47.6)	4487 (100)	
**Index date**				
Scores				
CHA_2_DS_2_-VASc index, mean (SD)	2.6 (1.6)	3.4 (1.2)	3 (1.5)	**<0.001**
HAS-BLED (SD), mean (SD)	2.8 (1)	3.5 (0.7)	3.1 (0.9)	**<0.001**
Concomitant medication				
Patients with concomitant medication, %	96.4%	99.3%	97.8%	**<0.001**
Concomitant medication, N (SD)	3.2 (1.6)	3.3 (1.4)	3.2 (1.5)	**0.029**
Percentage of use, N (%)				
NSAIDs	1559 (66.3)	1449 (67.8)	3008 (67.0)	0.278
Antiplatelet agents	953 (40.5)	898 (42.0)	1851 (41.3)	0.306
Antidiabetics	688 (29.3)	618 (28.9)	1306 (29.1)	0.807
Beta-blockers	1372 (58.4)	1269 (59.4)	2641 (58.9)	0.475
Agents acting in the renin–angiotensin system	1592 (67.7)	1495 (70)	3087 (68.8)	0.100
Lipid-lowering agent	1335 (56.8)	1290 (60.4)	2625 (58.5)	**0.014**
**End of the study**				
Scores				
CHA_2_DS_2_-VASc index, mean (SD)	2.7 (1.7)	3.6 (1.4)	3.1 (1.6)	**<0.001**
HAS-BLED (SD), mean (SD)	3.1 (1.2)	3.8 (0.9)	3.43 (1.1)	**<0.001**
Concomitant medication				
Patients with concomitant medication, %	96.4%	99.3%	97.8%	**<0.001**
Mean of concomitant medication, N (SD)	3.2 (1.6)	3.4 (1.4)	3.3 (1.5)	**0.002**
Percentage of use, N (%)				
NSAIDs	1577 (67.1)	1484 (69.5)	3061 (68.2)	0.085
Antiplatelet agents	970 (41.3)	921 (43.1)	1891 (42.1)	0.208
Antidiabetics	704 (29.9)	654 (30.6)	1358 (30.3)	0.624
Beta-blockers	1396 (59.4)	1305 (61.1)	2701 (60.2)	0.241
Agents acting in the renin–angiotensin system	1615 (68.7)	1526 (71.4)	3141 (70)	**0.045**
Lipid-lowering agents	1351 (57.5)	1325 (62)	2676 (59.6)	**0.002**
**Difference (end of the study—index date)**				
Scores				
CHA_2_DS_2_-VASc index, mean (SD)	0.1 (0.4)	0.2 (0.5)	0.2 (0.5)	**<0.001**
HAS-BLED (SD), mean (SD)	0.3 (0.5)	0.4 (0.59	0.3 (0.5)	**<0.001**
Concomitant medication, variation (SD)				
NSAID	0.8 (8.7)	1.6 (12.7)	1.2 (10.8)	**0.013**
*p*-value of variation	<0.001	<0.001	<0.001	
Antiplatelet agents	0.7 (8.5)	1.1 (10.3)	0.9 (9.4)	0.154
*p*-value of variation	<0.001	<0.001	<0.001	
Antidiabetics	0.7 (8.2)	1.7 (12.9)	1.2 (10.7)	**0.001**
*p*-value of variation	<0.001	<0.001	<0.001	
Beta-blockers	1 (10.1)	1.7 (12.9)	1.3 (11.5)	**0.041**
*p*-value of variation	<0.001	<0.001	<0.001	
Agents acting in the renin–angiotensin system	1 (9.8)	1.5 (12)	1.2 (10.9)	0.130
*p*-value of variation	<0.001	<0.001	<0.001	
Lipid-lowering agents	0.7 (8.2)	1.6 (12.7)	1.1 (10.6)	**<0.001**
*p*-value of variation	<0.001	<0.001	<0.001	

Data are expressed as mean values with standard deviations and percentages. Bold *p* values indicate statistical significance (*p* > 0.05). NSAID: Nonsteroidal anti-inflammatory drug; *p*: statistical significance; SD: standard deviation.

**Table 4:** Characteristics of the anticoagulant treatment.

Study Cohort	Adequate Anticoagulation Control	Poor Anticoagulation Control	Total	*p*-Value
N (%)	2351 (52.4)	2136 (47.6)	4487 (100)	
Time from diagnosis to first prescription				
Mean (SD)	13.2 (23.6)	12.5 (23)	12.8 (23.3)	0.352
Median (P25–P75)	1 (0–13)	1 (0–12)	1 (0–12)	
Treatment duration, days				
Mean (SD)	476.8 (262.8)	432.8 (259.4)	455.8 (262.1)	**<0.001**
Median (P25–P75)	576 (214–730)	355 (194–730)	461 (205–730)	
Medicine prescribed				
Use of acenocoumarol, N (%)	2121 (90.2)	1944 (91)	4065 (90.6)	0.338
Mean initial dose prescribed, mg (SD)	0.8 (0.7)	0.8 (0.7)	0.8 (0.7)	
Mean treatment duration, days (SD)	476.4 (263)	432.3 (260)	455.3 (263)	
Median treatment duration, days (P25–P75)	579.5 (213–730)	354 (194–730)	457.5 (205–730)	
Use of warfarin, N (%)	230 (9.8)	192 (9)	422 (9.4)	0.338
Mean initial dose prescribed, mg (SD)	1.3 (1.1)	1.3 (0.8)	1.3 (1)	
Mean treatment duration, days (SD)	480.4 (260.6)	436.9 (253.4)	460.6 (258)	
Median treatment duration, days (P25–P75)	564 (246–730)	376.5 (204–730)	465 (218–730)	
Discontinuation				
Patients for whom treatment was discontinued, N (%)	1321 (56.2)	1393 (65.2)	2719 (60.6)	**<0.001**
CI 95%	(54.2–58.2)	(63.2–67.2)	(59.2–62.0)	
Causes, N (%)	1322 (56.2)	1531 (71.7)	1425 (31.6)	**<0.001**
New events	487 (20.7)	722 (30.7)	600 (25.5)	
Medication switch	362 (15.4)	360 (15.3)	362 (15.4)	
Abandonment	315 (13.4)	214 (9.1)	268 (11.4)	
Mortality	158 (6.7)	235 (10)	195 (8.3)	
Persistence				
at 12 months, N (%)	1359 (57.8)	1053 (49.3)	2410 (53.7)	**<0.001**
CI 95%	(55.8–59.8)	(47.2–51.4)	(52.2–55.2)	
at 24 months, N (%)	1030 (43.8)	745 (34.9)	1772 (39.5)	**<0.001**
CI 95%	(41.8–45.8)	(32.9–36.9)	(38.1–40.9)	

Data are expressed as mean values with standard deviations and percentages. Bold *p* values indicate statistical significance (*p* > 0.05). CI: Confidence interval; *p*: statistical significance; P: percentile; SD: standard deviation.

**Table 5:** Use of healthcare resources.

Study Cohort	Adequate Anticoagulation Control	Poor Anticoagulation Control	Total	*p*-Value
N (%)	2351 (52.4)	2136 (47.6)	4487 (100)	
Visits				
Primary care visits, mean (SD)	15.8 (11.5)	16.6 (11.4)	16.2 (11.4)	**0.019**
Nurse visits, mean (SD)	14.3 (12.7)	17 (9.3)	15.6 (11.3)	**<0.001**
Specialist care visits, mean (SD)	2.1 (1.3)	2.2 (1.4)	2.2 (1.3)	**0.002**
Emergency visits, mean (SD)	0.8 (2)	0.8 (2.3)	0.8 (2.2)	0.203
Hospitalizations				
Mean (SD) number of hospitalizations	0.2 (0.7)	0.3 (0.7)	0.3 (0.7)	**0.006**
Hospitalization, N (%)	366 (15.6)	429 (20.1)	795 (17.7)	**<0.001**
Duration of hospitalizations, mean (SD) days,	1.7 (4.7)	2.6 (5.8)	2.1 (5.3)	**<0.001**
Tests, mean (SD)				
Laboratory	0.06 (0.46)	0.15 (0.76)	0.1 (0.62)	**<0.001**
Radiology	0.01 (0.14)	0.02 (0.16)	0.02 (0.15)	0.218
Computed tomography	0.01 (0.07)	0.01 (0.08)	0.01 (0.07)	0.659
Magnetic resonance	0 (0.04)	0 (0.04)	0 (0.04)	0.801
Catheterization	0 (0.03)	0 (0.03)	0 (0.03)	0.924
Angioplasty	0 (0.09)	0 (0.05)	0 (0.07)	0.648
Endarterectomy	0.01 (0.08)	0.01 (0.11)	0.01 (0.1)	0.120

Data are expressed as mean values with standard deviations and percentages. Bold *p* values indicate statistical significance (*p* > 0.05). *p*: statistical significance; SD: standard deviation.

**Table 6:** Healthcare costs (EUR, 2021).

Study Cohort	Adequate Anticoagulation Control	Poor Anticoagulation Control	Total	*p*-Value
N (%)	2351 (52.4)	2136 (47.6)	4487 (100)	
Visits, mean (SD)				
Primary care visits	367 (266)	385 (264)	376 (265)	**0.019**
Nurse visits	243 (215)	290 (158)	265 (192)	**<0.001**
Specialist care visits	193 (120)	204 (126)	198 (123)	**0.002**
Emergency visits	89 (238)	99 (268)	94 (253)	**0.203**
Hospitalizations	725 (1996)	1094 (2450)	901 (2231)	**<0.001**
Test, mean (SD)				
Laboratory	1 (10)	3 (17)	2 (14)	**<0.001**
Radiology	0 (0.3)	0 (0.3)	0 (0.3)	0.218
Computed tomography	0 (7)	1 (7)	1 (7)	0.659
Magnetic resonance	0 (7)	0 (7)	0 (7)	0.801
Catheterization	0 (5)	0 (6)	0 (6)	0.924
Angioplasty	1 (17)	1 (10)	1 (14)	0.648
Endarterectomy	1 (16)	2 (20)	2 (18)	0.120
Medication, mean (SD)				
Vitamin K antagonists	44 (32)	41 (30)	42 (31)	**0.007**
Concomitant medication	344 (206)	356 (182)	350 (195)	**0.045**
Total healthcare costs, mean (SD)	2010 (2103)	2477 (2554)	2232 (2340)	**<0.001**
			Difference *	
Total healthcare costs, mean **	2015	2470	−455	**<0.001**
95%CI	(1920–2109)	(2371–2569)		

* Differences in total healthcare costs of adequate control patients compared to those with poor control. ** Cost correction was performed using bootstrapping methodology. Data are expressed as mean values with standard deviations. Bold *p* values indicate statistical significance (*p* > 0.05). CI: confidence interval; *p*: statistical significance; SD: standard deviation.

## Data Availability

The data supporting this study are based on patient records, and no link is available; the information was aggregated upon study request. The data supporting this study’s findings are available on request from the corresponding author, A.S.M.

## Supplementary Materials

The following supporting information can be downloaded at https://www.mdpi.com/article/10.3390/jcm13216495/s1. Table S1: ICD-10-CM codes [31]; Table S2: CHA2DS2-VASc score for stroke risk in atrial fibrillation; Table S3: HAS-BLED score for bleeding risk on oral anticoagulation in atrial fibrillation; Table S4: Unit costs (year 2021) [30,37].
