# Supplementary material for: Consequences of the Poor Anticoagulation Control of Patients with Non-Valvular Atrial Fibrillation Treated with Vitamin K Antagonists
Source: J Clin Med. 2024 Oct 30;13(21):6495. doi: 10.3390/jcm13216495 (PMC11545910; doi:10.3390/jcm13216495)
Supplement: Supplementary file 1 [file jcm-13-06495-s001.zip › jcm-3276385-supplementary.pdf]

# Consequences of the Poor Anticoagulation Control of Patients with Non-Valvular Atrial Fibrillation Treated with Vitamin K Antagonists

**Antoni Sicras Mainar [1,*], Joel Salazar-Mendiguchía [2], María Isabel del Campo Alonso [2], Ainara Echeto [2], David Vilanova Larena [2] and Josep Comín Colet [3,4,5]**

1 Atrys Health, 28021 Madrid, Spain
2 Bristol Myers Squibb, 28050 Madrid, Spain; joel.salazar@bms.com (J.S.-M.); mariaisabel.campoalonso@bms.com (M.I.d.C.A.); ainara.echeto@bms.com (A.E.); david.vilanovalarena@bms.com (D.V.L.)
3 Cardiology Department, Bellvitge University Hospital, L'Hospitalet de Llobregat, 08907 Barcelona, Spain; josepcomin@gmail.com
4 IDIBELL—Instituto de Investigación Biomédica de Bellvitge, L'Hospitalet de Llobregat, 08908 Barcelona, Spain
5 CIBERCV—Centro de Investigación Biomédica en Red Enfermedades Cardiovaculares, Universitat de Barcelona, Hospitalet del Llobregat, 08007 Barcelona, Spain
* Correspondence: ihernandez@atryshealth.com; Tel.: +34-917-819-465

## Supplementary material

**Table S1.** ICD-10-CM codes. ........ 2
**Table S2.** $CHA_2DS_2$-VASc score for stroke risk in atrial fibrillation ........ 4
**Table S3.** HAS-BLED score for bleeding risk on oral anticoagulation in atrial fibrillation. ........ 5
**Table S4.** Unit costs (year 2021) ........ 6

**Table S1.** ICD-10-CM codes [31].

| Diagnoses | ICD-10-CM Codes |
|---|---|
| Atrial fibrillation, AF | I48.91 |
| Mitral valve heart disease | I05.0, I05.1, I05.2, I05.8, I08.0, I08.8, I08.9, I34.0, I34.8, Q20.0, Q20.1, Q20.3, Q20.4, Q20.5, Q20.8, Q21.0 - Q21.3, Q21.8, Q21.9, I27.83, Z95.2, Z95.3 |
| COVID-19 | B97.21 |
| ***Stroke or systemic embolism*** | |
| Ischemic stroke, transitory ischemic attack | I63.019, I63.119, I63.139, I63.20, I63.219, I63.22, I63.239, I63.30, I63.40, I63.50, I63.59, I67.89, G45.0, G45.1, G45.8, G45.9, I67.848 |
| Hemorrhagic stroke | I60.9, I61.9, I62.00, I62.1, I62.9 |
| Systemic embolism | I74.01, I74.09, I74.11, I74.2, I74.3, I74.5, I74.8, I74.9, I75.019, I75.029, I75.81, I75.89 |
| ***Major bleeding*** | |
| Intracranial bleeding | I60.9, I61.9, I62.1, I62.00, I62.9, S01.90XA, S06.4X0A, S06.4X1A, S06.4X2A, S06.4X3A, S06.4X4A, S06.4X5A, S06.4X6A, S06.4X7A, S06.4X8A, S06.4X9A, S06.5X0A, S06.5X1A, S06.5X2A, S06.5X3A, S06.5X4A, S06.5X5A, S06.5X6A, S06.5X7A, S06.5X8A, S06.5X9A, S06.6X0A, S06.6X1A, S06.6X2A, S06.6X3A, S06.6X4A, S06.6X5A, S06.6X6A, S06.6X7A, S06.6X8A, S06.6X9A, S01.90XA, S06.360A, S06.361A, S06.362A, S06.363A, S06.364A, S06.365A, S06.366A, S06.367A, S06.368A, S06.369A |
| Gastrointestinal bleeding | I85.01, I85.11, K22.11, K22.6, K22.8, K25.0, K56.699, K25.2, K25.4, K25.6, K26.0, K26.2, K26.4, K26.6, K27.0, K27.2, K27.4, K27.6, K28.0, K28.2, K28.4, K28.6, K29.01, K29.21, K29.41, K29.51, K29.61, K29.71, K29.81, K29.91, K52.81, K31.811, K31.82, K55.21, K57.11, K57.13, K57.31,K57.33, K62.5, K66.1, K92.0, K92.1, K92.2 |
| Bleeding from other sites | I31.2, M25.00, M25.019, M25.029, M25.039, M25.049, M25.059, M25.069, M25.073, M25.076, M25.08, N32.89, R04.1, R04.2, R04.9, R31.0, R58 |
| ***Others*** | |
| Venous thromboembolism (deep vein thrombosis and pulmonary embolism) | I26.90, I26.92, I26.99, I80.00, I80.10, I80.209, I80.219, I80.3, I80.8, I80.9, I81, I82.0, I82.1, I82.220, I82.221, I82.290, I82.291, I82.3, I82.409, I82.419, I82.429, I82.439, I82.449, I82.499, I82.4Y9, I82.4Z9, I82.509, I82.519, I82.529, I82.539, I82.549, I82.599, I82.5Y9, I82.5Z9, I82.609, I82.619, I82.629, I82.709, I82.719, I82.729, I82.819, I82.890, I82.891, I82.91, I82.A19, I82.A29, I82.B19, I82.B29, I82.C19, I82.C29, O22.3x, O22.9x, O87.1, O87.9, O88.219, O88.211, O88.212, O88.213, O88.22, O88.23, O88.819, O88.811, O88.812, O88.813, O88.82, O88.83, T80.0XXA, T81.718A, T81.72XA, T82.817A, T82.818A. |
| Cardiac surgery | Z95.2, Z95.3.<br>In addition, 116 codes regarding to procedures will be considered[37]. |
| Pericarditis | A06.89, A18.84, A39.53, B33.23, A52.06, A54.83, B39.9, I00, I01.0, I02.0, I09.2, I24.1, I30.0, I30.8, I30.9, I31.0, I31.1, I31.2, I31.8, I31.9, I32. |
| Hyperthyroidism and thyrotoxicosis | E05.xx |
| Pregnancy | O00.x, O01.9, O02.0, O02.1, O02.8x, O03.30 - O03.34, O03.37, O03.39, O03.8x, O03.9, O04.5 - O04.7, O04.8x, O07.x, O08.x, O09.00, O09.10, O09.A0, O09.291, 09.211, O09.30, O09.511, O09.611, O09.621, O09.819, O09.82x, O09.89x, O09.899, O09.9x, O09.4x, O09.5x, O10.x - O12.0x, O12.14, O12.15, O12.2x, O13.x - O16.x, O20.x - O22.x, O23.00, O23.10, O23.20, O23.30, O23.4x, O23.519, |

| Diagnoses | ICD-10-CM Codes |
| --- | --- |
| | O23.529, O23.599, O23.9x, O24.319, O24.32, O24.415, O24.419, O24.425, O24.429, O24.435, O24.439, O24.911 - O24.913, O24.92, O24.93, O25.x, O26.0x, O26.11 - O26.113, O26.2x, O26.41 - O26.43, O26.5x, O26.61x, O26.62, O26.81x - O26.85x, O26.87x, O26.89x, O26.90, O28.9, O30.00x, O30.02x, O30.10x, O30.20x, O30.80x, O30.9x, O31.00X0, O31.01X0, O31.02X0, O31.03X0, O31.10X0, O31.11X0, O31.3xX0, O31.8X10, O31.8X20, O31.8X30, O31.8X90, O32.xXX0, O33.0 - O33.2, O33.xXX0, O33.7XXx, O33.8, O33.9, O34.x, O35.xXX0, O36.01x0, O36.09x0, O36.11x0, O36.19x0, O36.4XX0, O36.51x0, O36.59x0, O36.6xX0, O36.80X0, O36.81x0, O36.82x0, O36.83x0, O36.89x0, O36.9xX0, O40.xXX0, O41.0xX0, O41.10x0, O41.12x0, O41.14x0, O41.8Xx0, O41.9xX0, O42.00, O42.011 - O42.013, O42.02, O42.10, O42.111 -O42.113, O42.12, O43.011, O43.019, O43.101 - O43.103, O43.199, O43.211 - O43.213, O43.221 - O43.223, O43.23x, O43.81x, O43.91 - O43.93, O44.x, O45.001 - O45.003, O45.011 - O45.013, O45.021 - O45.023, O45.091 - O45.093, O45.8Xx, O45.91 - O45.93, O46.x - O48.x, O60.0x, O60.1xX0, O61.0, O61.1, O61.9, O62.0 - O62.4, O62.9, O63.x, O64.0XX0, O64.1XX0, O64.9XX0, O65.4, O65.5, O65.9, O66.0, O66.1, O66.40 - O66.9, O67.x, O68, O69.xXX0, O69.8xX0, O69.9XX0, O70.x, O71.x (excl. O71.81), O72.x, O73.x, O74.1 - O74.3, O74.8, O74.9, O75.x, O76, O77.0, O8x (excl. O89.01), O89.3 - O89.6, O90.x, O91.01x, O91.02, O91.11x, O91.12, O91.21x, O91.22, O91.23, O92.01x, O92.03, O92.11x, O92.13, O92.2x, O92.3, O92.5 - O92.79, O94, O98.0x - O98.3x, O98.42, O98.43, O98.5x, O98.6x, O98.8x, O98.9x, O99.0x - O99.4x, O99.53, O99.63, O99.81x, O99.834, O99.835, O99.84x, O99.89, O9A.23, Z33.1, Z33.2, Z33.3, Z34.00, Z34.80, Z34.90, Z36.0, Z36.1, Z36.3 - Z36.5, Z36.8x, Z36.8A, Z36.9, Z37.0 - Z37.4, Z37.59, Z37.69, Z37.7, Z37.9, Z39.0, Z39.1, Z39.2, Z64.0.<br>In addition, 1058 codes regarding to procedures will be considered [31] |
| Hip and Knee Replacement Surgery | Z96.649, Z96.659, 0SQ90ZZ, 0SR90J9, 0SR90JA, 0SR90JZ, 0SRB0J9, 0SRB0JA, 0SRB0JZ.<br>In addition, 114 codes regarding to procedures will be considered [31] |

**Table S2.** $CHA_2DS_2$-VASc score for stroke risk in atrial fibrillation

| Feature | Score |
|---|---|
| Congestive heart failure | 1 |
| Hypertension | 1 |
| Age >75 years | 2 |
| Age 65-74 years | 1 |
| Stroke/TIA/TE | 2 |
| Vascular disease (previous MI, peripheral arterial disease, or aortic plaque) | 1 |
| Diabetes mellitus | 1 |
| Female | 1 |

MI, myocardial infarction; TE, systemic thromboembolism; TIA, transient ischemic attack.

**Table S3.** HAS-BLED score for bleeding risk on oral anticoagulation in atrial fibrillation.

| Feature | Score if present |
|---|---|
| Hypertension (systolic ≥160 mmHg) | 1 |
| Abnormal renal function | 1 |
| Abnormal liver function | 1 |
| Age >65 years | 1 |
| Previous stroke | 1 |
| Bleeding | 1 |
| Labile INRs | 1 |
| Taking concomitant drugs | 1 |
| Concomitant alcohol intake | 1 |

INR, international normalized ratio.

**Table S4.** Unit costs (year 2021)

| Health and non-health resources | Unit costs (€) |
|---|---|
| Medical visits | |
| Primary care medical visit | 23.19 |
| Specialized care medical visit* | 92.00 |
| Emergency medical visit | 117.53 |
| Hospitalization (one day) | 420.90 |
| Day hospital sessions** | 185.00 |
| Complementary tests | |
| Laboratory tests | 22.30 |
| Conventional radiology | 18.50 |
| Computed tomography | 96.00 |
| Magnetic nuclear resonance | 177.00 |
| Other diagnostic/therapeutic tests | 37.12 |
| Pharmaceutical prescription | Retail price |

Source of health resources: Sicras-Mainar *et al.,* 2019 [30] and INE [37].
*Healthcare and non-healthcare costs related to NVAF will be identified.
**Only in neurology, vascular, cardiology, internal medicine, geriatrics, endocrinology, and hematology services.
INE, National Institute of Statistics; NVAF, non-valvular atrial fibrillation.
